# Hospital and Institutional News

**Published:** 1921-02-12

**Authors:** 


### HOSPITAL AND INSTITUTIONAL NEWS.

### COMMITTEE ON VOLUNTARY HOSPITALS.

A further meeting of Lord Cave's Committee on Voluntary Hospitals was held on February 2, at the Ministry of Health, when evidence was given in the morning by Sir Cooper Perry and Mr. Maynard on behalf of the King Edward VII. Hospital Fund, and in the afternoon by Mr. Verity, Chairman of Charing Cross Hospital. Evidence was given later by Sir Samuel Scott, M.P., on behalf of the lying-in hospitals in the London Regional Area of the British Hospitals Association.

### A MAGNIFICENT ACHIEVEMENT.

Mr. Harry H. Woolley, J.P., the secretary of the Leicester and County Saturday Hospital Society, for the support of the Royal Infirmary and the Convalescent Homes, records a really magnificent achievement. The total of the Saturday Hospital Society's Fund for 1920 is now known, and it amounts to £32,666, compared with £21,631 in 1919. The increase, therefore, is one of £11,034. This remarkable progress is attributed to the operation of the resolution to raise the rate of weekly contributions from 1d. to 2d. We hope that this feat will be widely quoted. It is a triumph for the voluntary principle not only in Leicestershire but in the United Kingdom. Bravo! We commend the fact especially to the *Pall Mall Gazette*, which will doubtless welcome this example of the vitality of the voluntary principle.

### THE INFLUENZA REPORT.

It may be perfectly true that the Ministry's voluminous report on the Pandemic of Influenza contains very little new material, but the regrouping of the facts and their publication as a complete volume has given us a valuable addition to modern medical literature. From the public's essential viewpoint the report can hardly inspire confidence, in that the final solution is admittedly unachieved, but if they will but glance through its five hundred odd pages, its numberless diagrams, charts, and tables, they will realise the enormous amount of organised effort which even a provisional solution entails. And perhaps, also, they will then be more appreciative of the new Ministry's endeavours than certain of our lay contemporaries would apparently wish them to be. Many brief reviews of this report have already been published, but curiously little attention has been drawn to probably the most interesting chapter of all—namely, the history of influenza between 1658 and 1911. As a retrospect the report is excellent, and we can only hope that the future is receiving equally careful attention. (See p. 453.)

### INFLUENZA IN PAST TIMES.

Epidemic and even pandemic influenza have frequently occurred in the past. The sixteenth century epidemics known as the English sweats, where a tremendous case incidence was associated with low mortality, were probably influenzal. "Never," says a contemporary writer, "were so many people sick together, never did so few of them die." Sydenham records epidemics a little later characterised by severe cerebral and nervous manifestations, a predilection for the adult, but no serious effect upon the gross mortality for the year. Comatose types of fever are mentioned as frequent between 1673-5, and it is in many ways suggestive that case descriptions over that period run parallel with the apparent association of cerebro-spinal fever, influenza and encephalitis during the past few years. Later on, between 1728-52, four outbreaks of undoubted epidemic influenza ravaged this country. Again in May 1762 there was a typical explosion of the disease, reaching England from Copenhagen and extending to every province. But thereafter inferences are necessarily incomplete until 1847, ten years after the General Register Office had been established and Dr. Farr commenced the system destined to be the great exemplar of vital statistical methods. From this source it is ascertained that just prior to the 1847 pandemic the public health of the country was, as in 1918, at a low ebb, and when the outbreak came the death-rate increased about 34 per 1,000. But after this until 1889 this country was probably free from influenza for an exceptionally long period, and the statistical estimates of the disease ran relatively at their lowest. There were, indeed, in 1889, extremely few influenza deaths at all. Since 1890, however, this freedom has been gradually lost. 1891-2 were bad years with over 2,000 deaths; similarly 1895. Later the annual death-roll varied on either side of 1,000 until 1918, when it suddenly rose to 12,927!

### THE EFFICIENCY EXHIBITION.

As we go to press the so-called Efficiency Exhibition is opening at Olympia, and although one can as yet comment only upon the novelty of devoting such an organisation to an abstract quality, yet many of the spheres of national life which may in this way be dealt with are certain to appeal to readers of these pages. In our next issue we hope to discuss critically a number of these exhibits, which, judging from preliminary announcements, should give the intelligent worker, whatever his sphere, no little cause for reflection—and reform. The wide scope of the exhibition may be judged from the following summary of its sections: (*a*) Pure and applied scientific invention and research; (*b*) planning and equipment of factories, waste elimination, employees' welfare; (*c*) general commerce and institutional organisation; (*d*) national education and physique; (*e*) traffic and transport problems; (*f*) technical and professional training, vocational guidance, and many others. The only risk is that by covering so many fields the exhibition may lack finality in demonstration for any particular section. In any case there is one exhibit which we trust the public will duly appreciate, and this illustrates the Ministry of Labour's Industrial Training Department. The whole of the Olympia Annexe will be devoted to showing how the ex-fighting man is being trained to help also in the world-war of industry.

### ENCEPHALITIS LETHARGICA.

Although the returns throughout the country now show that little short of 150 cases per week of this disease have been recently notified, yet in this country, with a population of over forty millions, the view that nothing like a strong epidemic prevails is fully justified. Nevertheless, our imperfect knowledge concerning its pathology makes it satisfactory that official steps in the investigation are being taken. Among others, the Ministry of Health have written to the Lambeth Board of Guardians stating that the subject of encephalitis lethargica is at present engaging the attention of the medical officers of the Ministry, and it is desired that further laboratory investigation of this disease shall be undertaken. The Ministry points out that Dr. Pedrau, a part-time pathologist at the infirmary, has already rendered good service in connection with research work on pneumonia, and inquires whether the Guardians will be prepared to agree to the use of his services in the proposed inquiry, and to allow the laboratory facilities at the infirmary to be used by him for this purpose, the Ministry agreeing to defray all expenses. This is a valuable appreciation of the potentialities of such institutions, and we trust the experiment will be repeated.

### THE TUBERCULOSIS OUTLOOK.

One can only hope that the real inference to be drawn from the Registrar-General's report on tuberculosis in the country between 1911 and 1919 implies a genuine reduction of the disease. The most striking facts are (*a*) that in the last three quarters of 1919 there was apparently a reduction below the recent average mortality of some 10 to 20 per cent.; and (*b*) that the mortality below the age of five years has fallen in relation to both sexes. The first may be indirectly due to fewer acutely tuberculous subjects being left alive after the influenza epidemic. Both may arise from either increased bodily resistance or decrease in virulence of the disease. Probably the improved general conditions are chiefly responsible, and although the disease itself may be passing through a cycle in infective power, it is, in any case, premature to make too optimistic prophecies for the future. Remember, also, that the present adult figures refer to women alone.

### THE STAR AND GARTER MAGAZINE.

Because of the vicissitudes which have attended the transformation of the old Star and Garter Hotel since it was chosen, perhaps a little hastily, for a war hospital, its old patrons, its recent critics, and its new friends will read with interest the first number of the institution's new magazine, which appeared in January. The Hon. Sir Arthur Stanley, in an amusing letter to the editor, greets the paper as the "New Year's baby of journalism," and hopes, wisely, for "some idea of the inner life of the Home" in its pages. The editorial itself, happily, free from any apology, informs us that the paper will appear once a quarter, and that it is an offshoot of the "Sports and Games Club.' The staff seems to be versatile. Mr. Reginald Mundy, late of the Canadian Forces, has designed the cover, Mr. Mason provides a caricature, Mr. Lawrence North contributes a short story, Mr. Rudge Harding verse. It is delightful also to find a sympathetic review of Walter Pater's "Marius the Epicurean," which Mr. George Moore, whose "Avowals" contains the latest of his constant critical appreciations of the author, once described as "the golden book of English prose." Native talent seems abundant, and there are two fields for it to exploit in the Home at Richmond and at the branch at Sandgate, Kent, where, we may add, the editorial chair is situated. The British Red Cross Society, which received the Home from the Queen, to whom it had been presented in 1915 by the Auctioneers' and Estate Agents' Institute, has done much to encourage institutional journalism. The new magazine promises uncommonly well, and deserves indeed a longer review—it cannot receive a warmer welcome—than at the moment we have here given to it.

### MR. HARRIS CLEAVER'S SCHEME.

The latest attempt to use vacant and suitable accommodation in Poor-law infirmaries for the benefit of the middle classes has been made by Mr. H. P. Cleaver, and is attracting much attention, particularly in Manchester, where an ambitious plan on similar lines has already been noted. If Liverpool supports Mr. Cleaver with the same energy as Manchester is showing on its own lines, the West Derby Guardians should be successful. The fee proposed for private patients is three guineas or so a week. Accommodation is said to be readily available, and the scheme to be self-supporting from the start. The Liverpool hospitals are no less congested than those in Manchester, and are said to have pavilions equally suitable to the "auxiliaries" chosen for the Manchester scheme. The middle-class patient, as we all know, is now beset by many difficulties; his personal self-respect should be assured by Mr. Cleaver's plan, and we hope soon to learn that the Liverpool and Manchester proposals are running neck-to-neck to help him, and that their plans will be drawn with such consideration as to be a model and encouragement to others.

### NEW HOSPITAL FOR CARDIGAN.

The war memorial to the men of Cardigan is to be a hospital, for which the Priory, an historic house, has been bought for £4,250. Sir John Lynn Thomas has promised to give to the hospital not only all his instruments, but also his medical library and operating-table. A site for the hospital had previously been given by Sir Marteine Lloyd, Bart., and this is now being sold for the benefit of the hospital's funds. Cardigan itself has been chosen as one of the primary health centres under the Ministry of Health, and for this reason the future maintenance of the new hospital should be, in part at least, secured. The chairman of the hospital committee is Mr. John Evans, J.P.

### THE UNBORN CHILD.

There is no doubt that one of the secrets of improvement in the nation's health lies in the care of the unborn child, and it is in many ways satisfactory that the eighth and latest clinical unit of the Metropolitan medical schools should have been established in gynæcology and obstetrics, and this at the Royal Free Hospital, with its school of medicine for women. Apart from the fact that the establishment forms yet another recognition of the position that women have won in the medical profession, the workings of this unit should indirectly provide an invaluable basis for scientific improvement of the many pre-natal clinics already formed all over the country. The professorial chair will be occupied by a woman; the details of the appointment are to be announced at the next meeting of the Senate of London University.

### "A MEDICAL RAG."

There is much speculation as to exactly what the medical students of Guy's, Thomas's, Bart's and the London are going to do in the "turn," announced as "A Medical Rag," at the matinée at the London Coliseum on the 22nd instant, in aid of the National Hospital for the Paralysed and Epileptic. It is certainly very good of them to come to the assistance of a sister hospital in low water, and their performance is sure to sell many tickets. The "Rag" is divided into six "operations," and it is rumoured that in the grand finale there will be about 200 students on the stage with massed bands and mascots. We expect "The Venetian Blinds" from the London, with their drums and brasses, will materially increase the volume of sound. It is announced that H.M. the Queen will be present at this hospital matinée, and a very attractive programme has been announced. The residents' band at the London Hospital played a large part in the Christmas entertainments, and the "book of words" is given at full length in the *Gazette* for January. In a sketch entitled "The Rest Cure" an unfortunate patient, who simply wants to have a good sleep, is visited by a number of people, including all parts of the unit. The almoner wakes him up to remind him of his payment, and finally the chairman enters and announces that, through the failure of the appeal, "the hospital is closed this time"; the patient is then pulled out of bed and the bed wheeled away. In the final chorus the patients are informed that they will find "that Government treatment is not kind, we'll correspond all night, we'll correspond all day, by the time we've got his papers through, he will have passed away."

### TUBERCULOSIS APPOINTMENTS IN WALES.

A number of important appointments was made at the quarterly meeting on Friday of the Council of the Welsh National Memorial Association. Four candidates for the post of medical superintendent of the North Wales Sanatorium at Llangwyfan, Denbigh, vacated by Dr. D. B. Evans, on his acceptance of an appointment in Lancashire, appeared before the Council, and Mr. D. A. Powell, M.D., B.S. (Lond.), now tuberculosis physician for the counties of Anglesey and Carnarvon, was elected. Two other candidates—Mr. W. W. Fenwick Jones, M.B., B.S. (Lond.), now in charge of the tuberculosis department of University College Hospital, London, and acting as tuberculosis officer to the Metropolitan boroughs of Holborn and South St. Pancras, and Mr. Niven Robertson, M.B., Ch.B., D.P.H., M.D., medical superintendent of the National Sanatorium, Benenden, Kent—were appointed senior tuberculosis physicians. Dr. E. Falliott and Dr. Evan A. Mackenzie were appointed assistant tuberculosis physicians. Dr. Thirlwell Thomas, of Liverpool, was appointed honorary consulting surgeon to the Association, and Sir Robert Phillip, professor of tuberculosis, Edinburgh, honorary consulting physician. In addition, Mrs. Elizabeth Lloyd Jones was chosen matron of the Sealyham Hospital, Pembrokeshire, and Miss M. H. Ruffle as matron of the Cymla Hospital, Neath.

### FATAL ACCIDENT AT A HOSPITAL.

We much regret to learn that James Robinson, one of the men injured by the explosion of the boiler at the Royal Hospital for Diseases of the Chest, has since died. The accident took place on the morning of the 2nd instant at eight o'clock, and the engineer and three porters were injured, two of them being taken to St. Bartholomew's Hospital. Fortunately, such accidents are rare, and, pending the inquiry at the inquest, we do not attempt to speculate upon the circumstances of the explosion, but from the known facts it is clear that the boiler was not left unattended. The premises were considerably damaged and the fire brigade was called.

### SOME EARLY FINANCIAL STATEMENTS FOR 1920.

We have had in our hands for a fortnight or more proof sheets of the annual report of the Manchester Royal Infirmary, a fact which reflects credit on the accountants' department of such a large establishment. A satisfactory feature is the substantial increase of £3,495 in annual subscriptions, while patients' voluntary contributions, boxes and other headings also show good increases. Legacies were disappointing, amounting only to £7,097, and there was a deficit of £28,577 on the year. Ancoats Hospital also shows a balance on the wrong side—£7,339, although here also those responsible for getting in the funds have been far from idle. Subscriptions are £1,035 up and workmen's collections have doubled. Expenditure is again much heavier, but a rise of about forty per cent. under surgery and dispensary must have for it some abnormal reason. The Royal Infirmary, Sunderland, received £38,236 and paid £39,143; which in these times is quite a satisfactory result; here, too, there is a good increase under most income headings. Clayton Hospital and Wakefield General Dispensary for some reason run their accounts from June to June, and do not adopt the uniform system. All legacies and extraordinary income are funded, if they were treated as for current needs the accounts would show a surplus of £10,000 on the war years, but some of this money has been spent on extensions to the fabric of the hospital. The income of the Royal Westminster

Ophthalmic Hospital has risen from £4,339 in 1919 to £7,786 in 1920, and the expenditure from £5,648 to £8,172. The last figure includes a heavy bill for painting and repairs. The Committee expect to reduce the cost of maintenance in 1921 by about £1,000.

### SEPARATELY ADMINISTERED INFIRMARIES.

A conference of Poor-law Guardians in England whose infirmaries are under administration entirely separate from that of the Poor-law institution is to take place on February 17 at the offices of the City of London Union, 61 Bartholomew Close, E.C. The idea is to take time by the forelock and to be prepared with concerted action before the next Ministry of Health Bill appears. The Guardians of Ashton-under-Lyne Union have convened the meeting, holding the opinion that Guardians in the same position as themselves should be prepared to place their views before Parliament. One of the proposals to be brought forward at the conference is that surplus beds in Poor-law infirmaries, whether or not under separate administration, should be utilised for the ordinary public.

### A HARD CASE.

The Royal Albert Hospital, Devonport, has a case against the administrators of the National Relief Fund which appears to be reasonable. It has apparently been excluded from the list of grantees because, we are told, it possesses at the moment a small balance in hand. This, however, is said to be due to the sale of investments which were realised, to its own cost (now and in the future), in order to meet a heavy deficit. As the *Western Morning News* puts it, "in the case of a hospital, such a balance is no proof of future solvency." Indeed, the remedy of realisation was only a part of the financial disease. The totals of the latest balance sheet are, then, no satisfactory evidence of the real position, and the case is the harder because other hospitals, it is declared, with far larger invested funds—larger because their debts have not been paid—are understood to have received grants. For the audit of accounts moral as well as mathematical qualities are required, and the National Relief Fund should be asked if it has no suggestion for getting over a difficulty which is only technical. Being in its administration a "national" rather than a "voluntary" body, the Fund may prove unsympathetic and official. We hope not, because, if the facts are as stated, the responsibility for the present position rests really not with the hospital, but with the Fund.

### A PAPER OF IDEAS.

A paper which deserves to be read outside its place of origin is the *Bristol Medico-Chirurgical Journal.* Most of the new ideas, and there have been several, which have arisen on hospital questions at Bristol, either have been born, or at least nourished, in its pages. It took a prominent part, unless we are mistaken, in the recent controversy on hospital amalgamation there. In the main it seems to encourage centralisation, in the sense at least of active co-operation between the Bristol institutions. It also, we fancy, took an active share in the discussion of the best way to provide prompt accommodation for paying patients and for maternity cases in the city. This is all the more interesting from the fact that the title of the journal is an old one. We should be the last to wish that changed, for it is pleasant to remember how active a hospital centre Bristol was in the eighteenth century, and how full of ideas were the Smiths and other early friends of the Bristol Royal Infirmary. In the recent number the *Journal* attacks another hospital question of general interest—namely, the importance of "a properly equipped Almoner's Department, common to all the hospitals" (in Bristol). In a well-reasoned article the plan is defended because it would prevent abuse and afford a means of accommodating, by arrangement with the proper authority, such special types of patients as the ex-Service man suffering from a disability, whom without financial help the voluntary hospitals find it hard either to accept or to refuse. We note in the same article an explanation of the causes of the present financial difficulty. It is noteworthy that it is different from the one offered in the latest issue of "Burdett's Hospitals and Charities." As we said last week, no two people seem agreed. But we commend the article to our readers.

### MEDICAL COMFORTS AT NOMINAL PRICES.

We are pleased to note the progress of the work undertaken by the Priory for Wales of the Order of St. John. The transport service is working most effectively. Fifteen ambulance cars are in use, and these during the past twelve months have travelled 46,000 miles with 2,666 cases. Eleven stations have been established at various centres in the coalfield, and at a meeting of the executive committee of the Priory held at Cardiff last week it was decided to set up several others. Another phase of the Priory's work, writes a correspondent, is the maintenance of medical depôts, where large numbers of the general public have been able to obtain medical comforts at nominal prices.

### THIS WEEK'S DRUG MARKET.

The belief that the drug market is on the eve of improvement is still fairly prevalent, although it is difficult to find anything tangible to justify this. There is at present no increase in the volume of buying, while stocks of drugs, both home produced and imported, are accumulating. A revival in the export demand would make all the difference, but until the difficulties in the way of financing export trade are removed it is not easy to see what is going to bring about the revival. The downward movement of prices is much slower than it was a few months ago, and an inference may be that values in many instances have reached a level which yields only a small margin of profit. Camphor, cocaine, bromides, most of the synthetic drugs, a number of vegetable drugs and essential oils, like lemon and orange, have again eased off in price, but heavy reductions are rare. When buyers are convinced that the downward movement has ceased, business should be on a considerable scale, as stocks in the hands of retailers are said to be low.

